# The Frequencies of Gastroesophageal and Extragastroesophageal Symptoms in Patients with Mild Erosive Esophagitis, Severe Erosive Esophagitis, and Barrett's Esophagus in Taiwan

**DOI:** 10.1155/2013/480325

**Published:** 2013-08-12

**Authors:** Sung-Shuo Kao, Wen-Chih Chen, Ping-I Hsu, Seng-Kee Chuah, Ching-Liang Lu, Kwok-Hung Lai, Feng-Woei Tsai, Chun-Chao Chang, Wei-Chen Tai

**Affiliations:** ^1^Division of Gastroenterology, Department of Internal Medicine, Kaohsiung Veterans General Hospital, Kaohsiung, Taiwan; ^2^National Yang-Ming University, Taipei, Taiwan; ^3^Division of Hepato-Gastroenterology, Department of Internal Medicine, Kaohsiung Chang Gung Memorial Hospital and Chang Gung University College of Medicine, Kaohsiung City 833, Taiwan; ^4^Division of Gastroenterology, Department of Internal Medicine, Taipei Veterans General Hospital, Taipei, Taiwan; ^5^Department of Medical Education and Research, Kaohsiung Veterans General Hospital, Kaohsiung, Taiwan; ^6^Department of General Medicine, School of Medicine, College of Medicine, Taipei Medical University, Taipei, Taiwan

## Abstract

*Background*. Gastroesophageal reflux disease (GERD) may present with gastroesophageal and extraesophageal symptoms. Currently, the frequencies of gastroesophageal and extragastroesophageal symptoms in Asian patients with different categories of GERD remain unclear. *Aim*. To investigate the frequencies of gastroesophageal and extragastroesophageal symptoms in patients with mild erosive esophagitis, severe erosive esophagitis, and Barrett's esophagus of GERD. *Methods*. The symptoms of symptomatic subjects with (1) Los Angeles grade A/B erosive esophagitis, (2) Los Angeles grade C/D erosive esophagitis, and (3) Barrett's esophagus proven by endoscopy were prospectively assessed by a standard questionnaire for gastroesophageal and extragastroesophageal symptoms. The frequencies of the symptoms were compared by Chi-square test. *Result*. Six hundred and twenty-five patients (LA grade A/B: 534 patients; LA grade C/D: 37 patients; Barrett's esophagus: 54 patients) were assessed for gastroesophageal and extragastroesophageal symptoms. Patients with Los Angeles grade A/B erosive esophagitis had higher frequencies of symptoms including epigastric pain, epigastric fullness, dysphagia, and throat cleaning than patients with Los Angeles grade C/D erosive esophagitis. Patients with Los Angeles grade A/B erosive esophagitis also had higher frequencies of symptoms including acid regurgitation, epigastric acidity, regurgitation of food, nausea, vomiting, epigastric fullness, dysphagia, foreign body sensation of throat, throat cleaning, and cough than patients with Barrett's esophagus. *Conclusion*. The frequencies of some esophageal and extraesophageal symptoms in patients with Los Angeles grade A/B erosive esophagitis were higher than those in patients with Los Angeles grade C/D erosive esophagitis and Barrett's esophagus. The causes of different symptom profiles in different categories of GERD patients merit further investigations.

## 1. Introduction

The Montreal Definition and Classification of Gastroesophageal Reflux Disease defines GERD as a condition which develops when the reflux of stomach contents causes troublesome symptoms and/or complications [1]. Gastroesophageal reflux occurs when there is a transient decrease in tension in the lower esophageal sphincter, allowing gastric contents to leak into the esophagus [2]. In most people with GERD, gastric juice reflux causes heartburn, as a painful or burning sensation in the esophagus, but regurgitation of digestive juices is also common [3]. Other than two classic reflux symptoms above, dysphagia is reported by more than 30% of individuals with GERD [4]. Less common symptoms associated with GERD include water brash, burping, hiccups, nausea, and vomiting [5]. Gastroesophageal reflux may also be associated with manifestations affecting a wide range of extraesophageal tissues and organ systems. In the large German ProGERD study of patients presenting with heartburn, nearly one-third had extraesophageal reflux disorders at baseline. Common extraesophageal manifestations in GERD patients were chronic cough, laryngeal disorders, and asthma [6]. Some patients with GERD, however, are asymptomatic [7]. This is particularly true in the older adults, perhaps because of decreased acidity of the reflux material in some or decreased pain perception in others [8].

Although patients with Los Angeles grade C/D erosive esophagitis and Barrett's esophagus have more frequencies of acidic reflux episodes than those with LA grade A/B erosive esophagitis [9], the intensity and frequency of reflux symptoms are poor predictors of the presence of severe esophagitis. In a study investigating over 4000 patients with esophagitis, the percentage of patients with moderate or severe heartburn was comparable across all grades of disease [10]. Another study comparing the spectrum of heartburn severity in those with and without underlying esophagitis is similar, with over 60% of patients in both groups experiencing moderate or severe heartburn [11]. Additionally, an international, multicenter study revealed that the gastrointestinal symptom patterns were similar in patients with erosive and nonerosive esophagitis [12]. Another Chinese study also pointed out symptom resolution not predicting healing of erosive esophagitis [13]. These results may reflect the phenomenon that acid exposure is related to the severity of esophagitis but does not completely correlate with the severity of symptoms.

Barrett's esophagus, the normal squamous epithelium in the distal esophagus replaced by columnar epithelium, is considered one of the most important complications of gastroesophageal reflux disease [14]. There is controversy as to whether GERD exists as a spectrum of disease severity or as a categorical disease in three distinct groups, including Barrett's esophagus. In a prevalence study in Sweden, Barrett's esophagus was found in 1.6% of the general adult population, of which 56.3% had reflux symptoms [15]. Many patients with short-segment Barrett's esophagus have no GERD symptoms and no endoscopic signs of esophagitis in another study [16]. Bredenoord et al. discovered that patients with LA grade C/D reflux esophagitis and those with Barrett's esophagus have high total number of reflux episodes, but patients with LA grade C/D have higher percentage of reflux episodes reaching the proximal esophagus than those with Barrett's esophagus [9]. This might explain their low sensitivity to reflux in patients with Barrett's esophagus. 

Past studies regarding the prevalence of GERD symptoms were more focused on heartburn and acid regurgitation. There were no studies comparing the frequencies of all gastroesophageal and extragastroesophageal GERD symptoms in different severity of erosive esophagitis and Barrett's esophagus. In addition, the independent factors related to the development of extraesophageal symptoms remain unanswered. The aim of this study was therefore to compare the prevalence of gastroesophageal and extragastroesophageal symptoms in patients with various degrees of esophagitis and Barrett's esophagus. Special attention was also paid to the clinical factors related to the presence of extragastroesophageal symptoms.

## 2. Patients and Methods

### 2.1. Patients

Consecutive symptomatic patients with erosive esophagitis or histologically confirmed Barrett's esophagus diagnosed during endoscopy at Kaohsiung Veterans General Hospital and Kaohsiung Chang Gung Memorial Hospital of Taiwan between 2008 and 2012 were recruited. Subjects enrolled were further divided into three categories according to endoscopic findings: (1) mild erosive esophagitis: LA grade A/B erosive esophagitis, (2) severe erosive esophagitis: LA grade C/D erosive esophagitis, and (3) Barrett's esophagus. Patients were excluded if they had histories of (1) younger than 15 years old, (2) gastrointestinal malignancies, (3) pregnancy, (4) acute stress conditions (including sepsis, acute renal failure), (5) previous gastric surgery, (6) equivocal diagnosis of erosive esophagitis, and (7) taking proton pump inhibitor (PPI) and H2 receptor antagonist in the preceding 2 weeks before endoscopy. Baseline demographic data, smoking and alcohol histories were collected. 

### 2.2. Study Design

At the clinic visit, patients with acid regurgitation and/or heartburn were invited to receive panendoscopy surveillance for esophagitis or Barrett's esophagus. Patients with erosive esophagitis or Barrett's esophagus were prospectively assessed by a standard questionnaire for gastroesophageal and extragastroesophageal symptoms. All participants were asked about their consumption of H2-receptor antagonists and PPI over the past 2 weeks and about their tobacco, alcohol, coffee, and tea consumption. Venous blood samples for fasting glucose, cholesterol, and triglyceride were also taken. *Helicobacter pylori* infection was determined by the histology of gastric mucosa taken during endoscopy. 

#### 2.2.1. Definitions of Barrett's Esophagus and Erosive Esophagitis

At endoscopy, esophageal mucosal breaks (esophagitis) were graded from A to D according to the LA classification system [17, 18]. Esophageal biopsy was taken when salmon-pink mucosal projections from cardia were identified during endoscopy [19–21]. The diagnosis of Barrett's esophagus was confirmed by the presence of gastric or intestinal metaplasia in the esophageal biopsy specimens [22, 23].

#### 2.2.2. Questionnaire

A complete medical history and demographic data were obtained from each patient, including age, sex, body mass index (BMI), medical histories, and histories of smoking, alcohol, coffee, tea, spice, and sweets consumption. The history of gastroesophageal symptoms (including acid regurgitation, heartburn, epigastric acidity, bleeding, chest pain, regurgitation of food, nausea, vomiting, hiccup, epigastric pain, epigastric fullness, and dysphagia) and extraesophageal symptoms (including throat foreign body sensation, hoarseness, throat cleaning, cough, sore throat, and bad breath) were taken.

### 2.3. Statistics

Statistical analysis was performed using the Statistical Program for Social Sciences (SPSS 19.0 for windows). Univariate analysis was performed by Student's *t*-test for continuous variables and *χ* ^2^ test was used for categorical variables. Backward stepwise conditional binary logistic regression analysis was performed to determine independent risk factors of certain extragastroesophageal symptoms. *P* < 0.05 was considered statistically significant and all reported *P* values were two-sided.

## 3. Results

### 3.1. Study Population

Six hundred and twenty-five patients with erosive esophagitis or Barrett's esophagus were enrolled in the study. The mean age of the patients was 51.4 ± 12.4 years old, and 370 (59%) were males. They were categorized as mild erosive esophagitis (LA grade A/B; *n* = 534), severe erosive esophagitis (LA grade C/D, *n* = 37), and Barrett's esophagus (*n* = 54). Data regarding the clinical characteristics of patients at entry are summarized in (Table 1). Patients with LA grade C/D erosive esophagitis had higher mean age (56.89 ± 12.83 versus 51.05 ± 12.34), more male predominance (86.5% versus 56.2%), and more underlying hiatal hernia (70.3% versus 22.6%) than patients with LA grade A/B erosive esophagitis (Table 1). Additionally, they also had higher mean age (56.89 ± 12.83 versus 51.26 ± 11.66) and more underlying hiatal hernia (70.3% versus 27.8%) than patients with Barrett's esophagus. 

### 3.2. Frequencies of Gastroesophageal Symptoms in Different Categories of GERD

Table 2 lists the frequencies of gastroesophageal symptoms in each group of GERD patients. Generally, patients with mild (Los Angeles grade A/B) erosive esophagitis had more gastroesophageal symptoms. Patients with mild erosive esophagitis had higher frequencies of epigastric pain (50.4% versus 22.2%; *P* = 0.001), epigastric fullness (65.0% versus 43.2%; *P* = 0.008), and dysphagia (18.4% versus 5.4%; *P* = 0.045) than patients with severe erosive esophagitis. Patients with mild erosive esophagitis also had higher frequencies of acid regurgitation (86.3% versus 66.7%; *P* < 0.001), epigastric acidity (71.2% versus 51.9%; *P* = 0.003), regurgitation of food (32.6% versus 18.5%; *P* = 0.034), nausea (30.3% versus 16.7%; *P* = 0.035), vomiting (14.8% versus 1.9%; *P* = 0.008), epigastric fullness (65.0% versus 50.0%; *P* = 0.029), and dysphagia (18.4% versus 7.4%; *P* = 0.043) than patients with Barrett's esophagus. Additionally, patients with severe erosive esophagitis had higher frequency of acid regurgitation (89.2% versus 66.7%; *P* = 0.014) and vomiting (18.9% versus 1.9%; *P* = 0.005) than patients with Barrett's esophagus.

### 3.3. Frequencies of Extragastroesophageal Symptoms in Different Categories of GERD

Table 3 displays the frequencies of extragastroesophageal symptoms in each group of GERD patients. Patients with mild (Los Angeles grade A/B) erosive esophagitis had more frequent extragastroesophageal symptoms than the other two groups of patients. Patients with mild erosive esophagitis had higher frequency of throat cleaning (41.8% versus 21.6%; *P* = 0.016) than patients with severe erosive esophagitis. Patients with mild erosive esophagitis also had higher frequency of foreign body sensation of throat (50.5% versus 33.3%; *P* = 0.017), throat cleaning (41.8% versus 25.9%; *P* = 0.024), and cough (27.5% versus 14.8%; *P* = 0.043) than patients with Barrett's esophagus. In addition, cough was more frequent in patients with severe erosive esophagitis than patients with Barrett's esophagus (35.1% versus 14.8%; *P* = 0.024). 

### 3.4. Factors Related to the Presence of Extragastroesophageal Symptoms

Table 4 lists the independent factors of extragastroesophageal symptoms. We examined several possible variables for extragastroesophageal symptoms, such as age, gender, hiatal hernia, metabolic syndrome, and grade of esophagitis. The prevalence of foreign body sensation of throat was significantly higher in patients with mild erosive esophagitis (*P* = 0.031, odds ratio (OR): 2.039, and 95% confidence interval (CI): 1.067–3.899) (Table 4). For throat cleaning, mild erosive esophagitis was still the only independent factor contributing to prevalence (*P* = 0.037, OR: 2.077, and 95% CI: 1.044–4.133) (Table 4). Additionally, mild erosive esophagitis was an independent risk factor for the presence of cough (*P* = 0.037, OR: 2.575, and 95% CI: 1.058–6.272), while male gender was a protective factor (*P* = 0.019, OR: 0.618, and 95% CI: 0.414–0.923) for cough. We also found that patients with metabolic syndrome have lower rates of the development of sore throat (*P* = 0.034, OR: 0.574, and 95% CI: 0.343–0.960).

## 4. Discussion

This study is the first work simultaneously investigating the differences in gastroesophageal and extragastroesophageal symptoms among various categories of GERD. We have demonstrated that patients with LA grade A/B erosive esophagitis had higher frequencies of gastroesophageal symptoms (epigastric pain, epigastric fullness, and dysphagia) and extragastroesophageal symptoms (throat cleaning) than patients with LA grade C/D erosive esophagitis. In addition, they also had higher frequencies of gastroesophageal symptoms (acid regurgitation, epigastric acidity, regurgitation of food, nausea, vomiting, epigastric fullness, and dysphagia) and extragastroesophageal symptoms (foreign body sensation of throat, throat cleaning, and cough) than patients with Barrett's esophagus.

Our findings were consistent with a previous study reporting that patients with Barrett's esophagus had less frequent or less severe symptoms than patients with GERD [24]. Currently, the reasons for mild erosive esophagitis with more frequencies of gastroesophageal and extragastroesophageal symptoms remain unclear. Bredenoord et al., examining the episodes of all reflux, acid reflux, and weakly acid reflux in patients with different severity of GERD, showed that more reflux episodes were found in patients with more severe esophageal mucosal injury [9]. Another study also found that patients with erosive esophagitis had the longest duration of distal esophageal acid exposure than patients with nonerosive reflux disease and normal volunteers [25]. Therefore, the degree of acid exposure of esophagus cannot explain the findings in our study. Possible explanations for our findings include different esophageal sensitivity and different frequencies of laryngopharyngeal reflux in various categories of GERD. We suppose that the esophageal mucosa in patients with mild erosive esophagitis may be more sensitive to refluxate than patients with severe erosive esophagitis or Barrett's esophagus. Second, laryngopharyngeal reflux is different in each group of GERD patients. Bredenoord et al. reported that patients with Barrett's esophagus having fewer reflux episodes reached proximal esophagus when compared with patients of Los Angeles grade C/D erosive esophagitis [9]. The finding may explain lower frequency of extragastroesophageal symptoms in patients with Barrett's esophagus than in patients with severe erosive esophagitis.

In this study, we also searched for independent risk factors related to the presence of extragastroesophageal symptoms. Mild erosive esophagitis was identified as a risk factor for extragastroesophageal symptoms including foreign body sensation of throat, throat cleaning, and cough. Male gender was identified as a negative factor for cough symptom and metabolic syndrome as a negative factor for sore throat. In previous ProGERD study [6], female gender, old age, severity of erosive reflux disease, duration of GERD, and smoking were identified as risk factors for the occurrence of extraoesophageal disorders. 

Our study has several limitations. The true prevalence of extragastroesophageal symptoms is difficult to determine because it is difficult to evaluate whether GERD is the cause of extragastroesophageal condition or whether the two conditions coexist independently of each other [26]. Secondly, patients with milder symptoms may take medicine over the counter, making study groups to be more highly selective. Third, the lack impedance-pH monitor and symptom correlation limited our hypothesis to the current finding.

In conclusion, the frequencies of some esophageal and extraesophageal symptoms in patients with Los Angeles grade A/B erosive esophagitis were higher than those in patients with Los Angeles grade C/D erosive esophagitis and Barrett's esophagus. The causes of different symptom profiles in different categories of GERD patients merit further investigations.

## Figures and Tables

**Table 1:** Demographic data of patients with mild erosive esophagitis, severe esophagitis, and Barrett's esophagus.

Characteristics	Mild erosive	Severe erosive	Barrett's
	esophagitis	esophagitis	esophagus
	(LA grade A/B)	(LA grade C/D)	
Patient number	*N* = 534	*N* = 37	*N* = 54
Age (yr) (mean ± SD)	51.05 ± 12.34	56.89 ± 12.83*	51.26 ± 11.66^#^
Gender (male)	300/534 (56.2%)	32/37 (86.5%)*	38/54 (70.4%)
Metabolic syndrome	144/458 (31.4%)	15/35 (42.9%)	15/46 (32.6%)
Hiatal hernia	120/532 (22.6%)	26/37 (70.3%)*	15/54 (27.8%)^#^

*P < 0.05 compared with esophagitis A/B.

^#^ *P* < 0.05 compared with esophagitis C/D.

**Table 2:** Frequencies of gastroesophageal symptoms in patients with mild erosive esophagitis, severe erosive esophagitis, and Barrett's esophagus.

Symptoms	Mild erosive	Severe erosive	Barrett's
	esophagitis	esophagitis	esophagus
	(LA grade A/B)	(LA grade C/D)	
Acid regurgitation	461/534 (86.3%)	33/37 (89.2%)	36/54 (66.7%)^∗#^
Heartburn	312/534 (58.4%)	18/37 (48.6%)	27/54 (50.0%)
Epigastric acidity	380/534 (71.2%)	21/37 (56.8%)	28/54 (51.9%)*
Esophageal bleeding	11/533 (2.1%)	2/37 (5.4%)	0/54 (0.0%)
Chest pain	177/466 (38.0%)	13/37 (35.1%)	14/54 (25.9%)
Regurgitation of food	152/466 (32.6%)	7/37 (18.9%)	10/54 (18.5%)*
Nausea	162/534 (30.3%)	7/37 (18.9%)	9/54 (16.7%)*
Vomiting	79/534 (14.8%)	7/37 (18.9%)	1/54 (1.9%) ^∗#^
Hiccup	289/534 (54.1%)	16/37 (43.2%)	26/54 (48.1%)
Epigastric pain	269/534 (50.4%)	8/36 (22.2%)*	20/54 (37.0%)
Epigastric fullness	347/534 (65.0%)	16/37 (43.2%)*	27/54 (50.0%)*
Dysphagia	98/534 (18.4%)	2/37 (5.4%)*	4/54 (7.4%)*

*P < 0.05 compared with esophagitis A/B.

^#^ *P* < 0.05 compared with esophagitis C/D.

**Table 3:** Frequencies of extragastroesophageal symptoms in patients with mild erosive esophagitis, severe erosive esophagitis, and Barrett's esophagus.

Symptoms	Mild erosive	Severe erosive	Barrett's
	esophagitis	esophagitis	esophagus
	(LA grade A/B)	(LA grade C/D)	
Foreign body sensation of throat	236/467 (50.5%)	13/37 (35.1%)	18/54 (33.3%)*
Hoarseness	164/534 (30.7%)	12/37 (32.4%)	11/54 (20.4%)
Throat cleaning	195/466 (41.8%)	8/37 (21.6%)*	14/54 (25.9%)*
Cough	147/534 (27.5%)	13/37 (35.1%)	8/54 (14.8%)^∗#^
Sore throat	102/534 (19.1%)	6/37 (16.2%)	9/54 (16.7%)

*P < 0.05 compared with esophagitis A/B.

^#^ *P* < 0.05 compared with esophagitis C/D.

**Table 4:** Independent factors for extragastroesophageal symptoms in patients with mild erosive esophagitis, severe erosive esophagitis, and Barrett's esophagus.

Symptoms	Risk factors	Coefficient	Standard error	OR (95% CI)	*P* value
Foreign body sensation	Esophagitis A/B	0.713	0.331	2.039 (1.067–3.899)	0.031
Throat cleaning	Esophagitis A/B	0.731	0.351	2.077 (1.044–4.133)	0.037
Cough	Esophagitis A/B	0.946	0.454	2.575 (1.058–6.272)	0.037
	Male gender	−0.481	0.204	0.618 (0.414–0.923)	0.019
Sore throat	Metabolic syndrome	−0.555	0.263	0.574 (0.343–0.960)	0.034
Hoarseness*	N/A	N/A	N/A	N/A	N/A

*No single risk factor was identified contributing to the development of hoarseness.
